# Multiple Stafne Bone Defects: A Rare Entity

**DOI:** 10.5402/2011/792145

**Published:** 2011-02-06

**Authors:** Luciana Barreto Vieira Aguiar, Frederico Sampaio Neves, Luana Costa Bastos, Iêda Crusoé-Rebello, Glaucia Maria Bovi Ambrosano, Paulo Sérgio Flores Campos

**Affiliations:** ^1^Department of Oral Radiology, Piracicaba Dental School, State University of Campinas, P.O. Box 52, 13414-903 Piracicaba, SP, Brazil; ^2^Department of Oral Radiology, School of Dentistry, Federal University of Bahia, Rua Araújo Pinho, 62, Salvador–BA 40110-150, Brazil

## Abstract

Stafne bone defects (SBDs) are generally located in the lingual cortex, close to the mandibular angle. We report the occurrence of multiple SBDs in an asymptomatic patient, a 60-year-old man, referred to a radiology clinic to undergo examination for the purpose of implant planning. The case of multiple SBD presented here, probably the first reported in the literature, reinforces the hypothesis that some cases of SBD may be the result of a focal failure during the ossification of the mandible.

## 1. Introduction

Stafne bone defect (SBD), first described in 1942 [1], is a lingual bone cavity with an evident cortical contour at the second/third molar region, below the mandibular canal and anterior to the mandibular angle. It has a circular or oval shape (1–3 cm in diameter), and when it is oval, the greater axis is parallel to the inferior edge of the mandible. It affects the male gender to a significantly greater extent (70%–90%) and appears more frequently in the fifth and sixth decades of life [1–3].

In addition to occurring more commonly in the posterior region of the mandible (posterior variant), SBD may also appear in the anterior region (anterior variant) and in the ascending ramus of the mandible (mandibular ramus variant) [2]. Normally it is unilateral, with some reports of bilateral occurrence [1, 4, 5]. Double unilateral occurrence is rare, and bilocular occurrence is even rarer [3].

It has been suggested that in a large number of cases of SBD the causal factor is mechanical pressure caused by the glandular tissue surrounding the lingual cortex of the mandible. This theory has been accepted because the radiolucent areas found on radiographs are associated with the submandibular and sublingual glands [3].

Normal salivary gland tissue is the most common histological finding, suggesting a developmental origin in which a part of the submandibular gland was trapped in the lingual mandibular aspect [4]. However, exploratory procedures on bone defects have shown that in a minority of cases, muscular, fibrous vascular, adipose, or lymphoid tissue may also be associated [5].

This paper presents a rare case of multiple bone defects, one posterior and two anterior variants, confirmed by computed tomography.

## 2. Case Report

**Figure 1:**
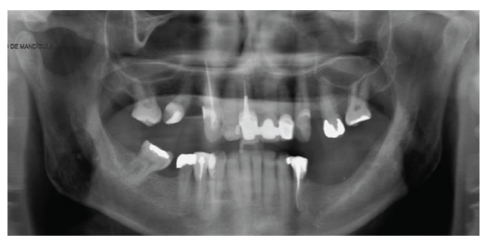
Panoramic radiograph did not reveal bone defects in the mandible.

The asymptomatic patient, a 60-year-old man, was referred to a radiology clinic to undergo examination for the purpose of implant planning. The panoramic radiograph revealed no important alterations in the mandible (Figure 1). The patient underwent multidetector computed tomography (MDCT) (Synergy Helicoidal, General Electric Company, Milwaukee, WI), with slices and intervals of 0.625 mm, field of view 15.8 cm, matrix 512 × 512, standard filter, 120 kV and 200 mA.

**Figure 2:**
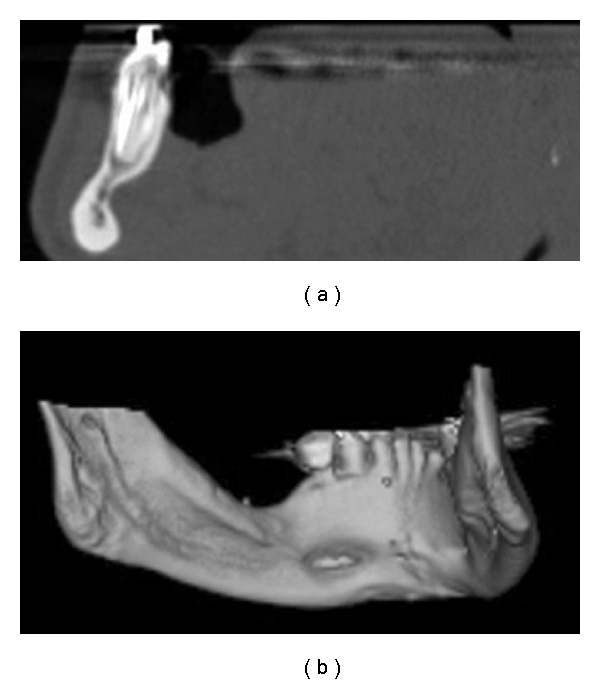
Sagittal computed tomography slices and bone window (a) and 3D reconstruction (b), showing SBD, anterior variant, left side.

**Figure 3:**
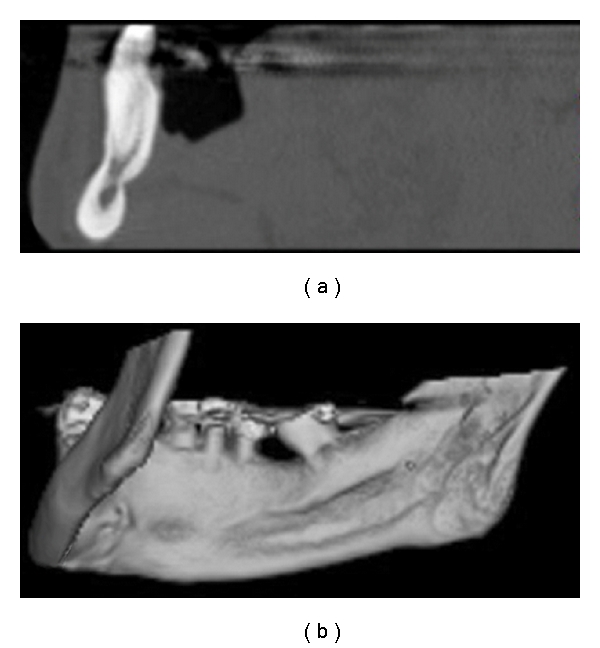
Sagittal computed tomography slices and bone window (a) and 3D reconstruction (b), showing SBD, anterior variant, right side, with small depth.

**Figure 4:**
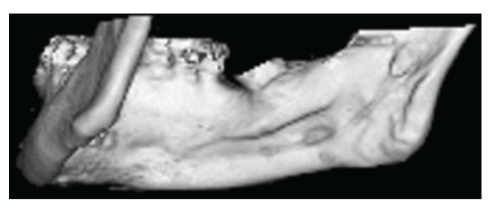
Volume-rendering 3D reconstruction showing a shallow SBD, posterior variant, on the right side.

The tomographic images clearly showed multiple cavitary defects, two in the anterior region (anterior variant) and one in the posterior region (posterior variant) (Figures 2, 3, and 4). Intracavitary tomographic density (Hounsfield units) suggested glandular tissue in the anterior defects and fat in the posterior defect.

## 3. Discussion

Stafne [1] initially suggested that these cavitary defects resulted from failures in the ossification process in areas of the Meckel cartilage.

Philipsen et al. [2] affirmed that SBD is formed through the pressure exerted by hyperplastic or hypertrophic salivary glands on the bone surface. Campos et al. [6] showed that the mandibular ramus variant does not have such an origin, because the parotid gland is not in contact with the lingual aspect of the ascending ramus of the mandible, which is covered by the medial pterygoid muscle.

Minowa et al. [7] considered that SBD, posterior variant, is the result of erosion caused by an acquired vascular lesion. Campos [8], however, considered that vascular pressure would contribute to the development of the defect but that the main cause of the SBD posterior variant formation is hypertrophy/hyperplasia of the submandibular gland. Only a limited number of cases would be the result of defective bone formation.

Bone erosion caused by lipoma is another causative factor hypothesized by Minowa et al. [7]. This hypothesis is supported by the fact that this condition is a rare occurrence in childhood in the region of the head and neck, which would justify the absence of SBD in this age group [9].

For the case under discussion, we believe that the filling of the cavities with glandular and fat tissue shows that the neighboring tissues tend to occupy the bone defect, despite the fact that in most cases, the relationship between SBD and the salivary gland is unquestionable. In our opinion, the multiple occurrence of SBD described here, characterized by no evidence of cortex erosion and small-depth depression, confirms that in some cases, SBD may be the result of a focal failure of mandible ossification.
